# Opipramol

**DOI:** 10.1107/S1600536811021131

**Published:** 2011-06-11

**Authors:** Hoong-Kun Fun, Wan-Sin Loh, M. S. Siddegowda, H. S. Yathirajan, B. Narayana

**Affiliations:** a X-ray Crystallography Unit, School of Physics, Universiti Sains Malaysia, 11800 USM, Penang, Malaysia; b Department of Studies in Chemistry, University of Mysore, Manasagangotri, Mysore 570 006, India; c Department of Studies in Chemistry, Mangalore University, Mangalagangotri 574 199, India

## Abstract

In the title compound (systematic name: 2-{4-[3-(5*H*-dibenz[*b*,*f*]azepin-5-yl)prop­yl]piperazin-1-yl}ethanol), C_23_H_29_N_3_O, the 5*H*-dibenz[*b*,*f*]azepine and piperazine rings adopt boat and chair conformations, respectively, and the overall shape of the fused ring part of the molecule is a butterfly. In the crystal, O—H⋯N and C—H⋯O hydrogen bonds link the mol­ecules into a layer parallel to the *bc* plane.

## Related literature

For the application of opipramol, see: Moller *et al.* (2001 ▶). For related structures, see: Jasinski *et al.* (2010 ▶); Nagaraj *et al.* (2005 ▶). For ring conformations, see: Cremer & Pople (1975 ▶). For bond-length data, see: Allen *et al.* (1987 ▶). For the stability of the temperature controller used for the data collection, see: Cosier & Glazer (1986 ▶).
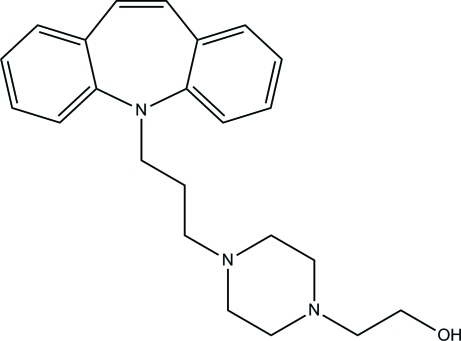


## Experimental

### 

#### Crystal data

C_23_H_29_N_3_O
*M* *_r_* = 363.49
Monoclinic, 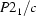

*a* = 12.6215 (2) Å
*b* = 10.5929 (2) Å
*c* = 14.3629 (2) Å
β = 90.966 (1)°
*V* = 1920.02 (5) Å^3^
*Z* = 4
Mo *K*α radiation
μ = 0.08 mm^−1^
*T* = 100 K
0.59 × 0.36 × 0.30 mm

#### Data collection

Bruker SMART APEXII CCD area-detector diffractometer
Absorption correction: multi-scan (*SADABS*; Bruker, 2009 ▶) *T*_min_ = 0.955, *T*_max_ = 0.977
30830 measured reflections
7949 independent reflections
6682 reflections with *I* > 2σ(*I*)
*R*_int_ = 0.026

#### Refinement

*R*[*F* ^2^ > 2σ(*F* ^2^)] = 0.042
*wR*(*F* ^2^) = 0.117
*S* = 1.03
7949 reflections
248 parameters
H atoms treated by a mixture of independent and constrained refinement
Δρ_max_ = 0.56 e Å^−3^
Δρ_min_ = −0.25 e Å^−3^

### 

Data collection: *APEX2* (Bruker, 2009 ▶); cell refinement: *SAINT* (Bruker, 2009 ▶); data reduction: *SAINT*; program(s) used to solve structure: *SHELXTL* (Sheldrick, 2008 ▶); program(s) used to refine structure: *SHELXTL*; molecular graphics: *SHELXTL*; software used to prepare material for publication: *SHELXTL* and *PLATON* (Spek, 2009 ▶).

## Supplementary Material

Crystal structure: contains datablock(s) global, I. DOI: 10.1107/S1600536811021131/is2726sup1.cif

Structure factors: contains datablock(s) I. DOI: 10.1107/S1600536811021131/is2726Isup2.hkl

Supplementary material file. DOI: 10.1107/S1600536811021131/is2726Isup3.cml

Additional supplementary materials:  crystallographic information; 3D view; checkCIF report

## Figures and Tables

**Table 1:** Hydrogen-bond geometry (Å, °)

*D*—H⋯*A*	*D*—H	H⋯*A*	*D*⋯*A*	*D*—H⋯*A*
O1—H1O1⋯N2^i^	0.896 (16)	1.999 (16)	2.8822 (9)	168.3 (14)
C5—H5*A*⋯O1^ii^	0.95	2.41	3.3478 (11)	167

Symmetry codes: (i) 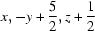; (ii) 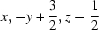.

**Fig. 1.**
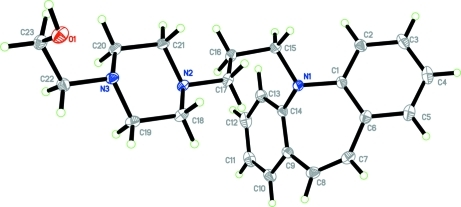
The molecular structure of the title compound, showing 50% probability displacement ellipsoids and the atom-numbering scheme.

**Fig. 2.**
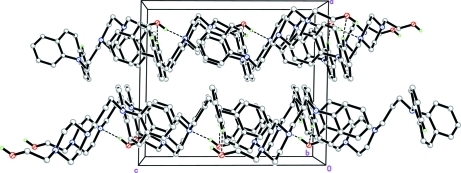
The crystal packing of the title compound, viewed along the b axis. H atoms not involved in the intermolecular interactions (dashed lines) have been omitted for clarity.

## supplementary crystallographic information

### Comment

Opipramol [systematic IUPAC name: 4-[3-(5*H*-dibenz[*b*,*f*] azepin-5-yl)propyl]-1-piperazinethanol] is an antidepressant and anxiolytic typically used in the treatment of generalized anxiety disorder (Moller *et al.*, 2001). Opipramol is a tricyclic compound with no reuptake-inhibiting properties. However, it has pronounced D2-, 5-HT2-, and H1-blocking potential and high affinity to sigma receptors (sigma-1 and sigma-2). The crystal structure studies of opipramol dipicrate is reported (Jasinski *et al.*, 2010). In view of the importance of the title compound, C_23_H_29_N_3_O, the crystal structure is reported.

In the title compound (Fig. 1), the seven-membered, 5*H*-dibenz[*b*,*f*]azepine ring (N1/C1/C6–C9/C14) adopts a boat conformation (Cremer & Pople, 1975) and the overall molecular shape is that of a butterfly (Nagaraj *et al.*, 2005) whereas the piperazine ring (N2/C18/C19/N3/C20/C21) adopts a chair conformation (Cremer & Pople, 1975) with the puckering parameters, Q = 0.5934 (8) Å, Θ = 177.94 (8)°, φ = 305.8 (18)°. The torsion angle between the 5*H*-dibenz[*b*,*f*]azepine and the piperazine rings, N1–C15–C16–C17 is 61.73 (8)°. Bond lengths (Allen *et al.*, 1987) and angles are within the normal ranges.

In the crystal packing (Fig. 2), intermolecular O1—H1O1···N2 and C5—H5A···O1 hydrogen bonds link the molecules into layers parallel to the *bc* plane.

### Experimental

The title compound was obtained as a gift sample from *R. L*. Fine Chem. Ltd., Bangalore, India. The compound was recrystallized from acetone (*m. p.* 373–374 K).

### Refinement

H1O1 was located from a difference map and was refined freely [O—H = 0.896 (16) Å]. The remaining H atoms were positioned geometrically (C—H = 0.95 or 0.99 Å) and refined with a riding model with *U*_iso_(H) = 1.2*U*_eq_(C).

### Crystal data

**Table:**

C_23_H_29_N_3_O	*F*(000) = 784
*M**_r_* = 363.49	*D*_x_ = 1.257 Mg m^−^^3^
Monoclinic, *P*2_1_/*c*	Mo *K*α radiation, λ = 0.71073 Å
Hall symbol: -P 2ybc	Cell parameters from 9885 reflections
*a* = 12.6215 (2) Å	θ = 2.9–34.2°
*b* = 10.5929 (2) Å	µ = 0.08 mm^−^^1^
*c* = 14.3629 (2) Å	*T* = 100 K
β = 90.966 (1)°	Block, colourless
*V* = 1920.02 (5) Å^3^	0.59 × 0.36 × 0.30 mm
*Z* = 4	

### Data collection

**Table:**

Bruker SMART APEXII CCD area-detector diffractometer	7949 independent reflections
Radiation source: fine-focus sealed tube	6682 reflections with *I* > 2σ(*I*)
graphite	*R*_int_ = 0.026
φ and ω scans	θ_max_ = 34.3°, θ_min_ = 2.4°
Absorption correction: multi-scan (*SADABS*; Bruker, 2009)	*h* = −16→19
*T*_min_ = 0.955, *T*_max_ = 0.977	*k* = −13→16
30830 measured reflections	*l* = −22→20

### Refinement

**Table:**

Refinement on *F*^2^	Primary atom site location: structure-invariant direct methods
Least-squares matrix: full	Secondary atom site location: difference Fourier map
*R*[*F*^2^ > 2σ(*F*^2^)] = 0.042	Hydrogen site location: inferred from neighbouring sites
*wR*(*F*^2^) = 0.117	H atoms treated by a mixture of independent and constrained refinement
*S* = 1.03	*w* = 1/[σ^2^(*F*_o_^2^) + (0.0611*P*)^2^ + 0.4097*P*] where *P* = (*F*_o_^2^ + 2*F*_c_^2^)/3
7949 reflections	(Δ/σ)_max_ = 0.001
248 parameters	Δρ_max_ = 0.56 e Å^−^^3^
0 restraints	Δρ_min_ = −0.25 e Å^−^^3^

### Special details

**Table:**

Experimental. The crystal was placed in the cold stream of an Oxford Cryosystems Cobra open-flow nitrogen cryostat (Cosier & Glazer, 1986) operating at 100.0 (1) K.
Geometry. All e.s.d.'s (except the e.s.d. in the dihedral angle between two l.s. planes) are estimated using the full covariance matrix. The cell e.s.d.'s are taken into account individually in the estimation of e.s.d.'s in distances, angles and torsion angles; correlations between e.s.d.'s in cell parameters are only used when they are defined by crystal symmetry. An approximate (isotropic) treatment of cell e.s.d.'s is used for estimating e.s.d.'s involving l.s. planes.
Refinement. Refinement of *F*^2^ against ALL reflections. The weighted *R*-factor *wR* and goodness of fit *S* are based on *F*^2^, conventional *R*-factors *R* are based on *F*, with *F* set to zero for negative *F*^2^. The threshold expression of *F*^2^ > σ(*F*^2^) is used only for calculating *R*-factors(gt) *etc*. and is not relevant to the choice of reflections for refinement. *R*-factors based on *F*^2^ are statistically about twice as large as those based on *F*, and *R*- factors based on ALL data will be even larger.

### Fractional atomic coordinates and isotropic or equivalent isotropic displacement parameters (Å^2^)

**Table:**

	*x*	*y*	*z*	*U*_iso_*/*U*_eq_	
O1	0.10636 (5)	1.33255 (6)	1.06904 (4)	0.01965 (12)	
N1	0.33738 (5)	0.74218 (6)	0.57588 (4)	0.01418 (11)	
N2	0.21096 (5)	1.06560 (6)	0.73239 (4)	0.01326 (11)	
N3	0.12890 (5)	1.23458 (6)	0.87387 (4)	0.01380 (11)	
C1	0.35654 (6)	0.60944 (7)	0.58110 (5)	0.01411 (12)	
C2	0.45637 (6)	0.56231 (8)	0.60674 (5)	0.01763 (13)	
H2A	0.5136	0.6192	0.6165	0.021*	
C3	0.47316 (7)	0.43319 (8)	0.61812 (5)	0.02086 (15)	
H3A	0.5412	0.4027	0.6363	0.025*	
C4	0.39006 (8)	0.34908 (8)	0.60285 (6)	0.02318 (16)	
H4A	0.4011	0.2610	0.6106	0.028*	
C5	0.29077 (7)	0.39456 (8)	0.57628 (6)	0.02090 (15)	
H5A	0.2344	0.3367	0.5656	0.025*	
C6	0.27213 (6)	0.52452 (7)	0.56485 (5)	0.01608 (13)	
C7	0.16454 (6)	0.56539 (8)	0.53991 (5)	0.01885 (14)	
H7A	0.1084	0.5136	0.5611	0.023*	
C8	0.13636 (6)	0.66812 (8)	0.49038 (5)	0.01869 (14)	
H8A	0.0627	0.6846	0.4831	0.022*	
C9	0.20930 (6)	0.75680 (7)	0.44664 (5)	0.01590 (13)	
C10	0.17851 (7)	0.81058 (8)	0.36112 (5)	0.02081 (15)	
H10A	0.1099	0.7931	0.3362	0.025*	
C11	0.24589 (8)	0.88867 (8)	0.31209 (5)	0.02329 (16)	
H11A	0.2239	0.9232	0.2539	0.028*	
C12	0.34570 (7)	0.91589 (8)	0.34884 (6)	0.02238 (16)	
H12A	0.3926	0.9685	0.3153	0.027*	
C13	0.37721 (6)	0.86616 (8)	0.43480 (5)	0.01829 (14)	
H13A	0.4454	0.8858	0.4597	0.022*	
C14	0.30944 (6)	0.78756 (7)	0.48477 (5)	0.01414 (12)	
C15	0.40721 (6)	0.82280 (7)	0.63287 (5)	0.01655 (13)	
H15A	0.4750	0.8347	0.6004	0.020*	
H15B	0.4229	0.7799	0.6928	0.020*	
C16	0.35831 (6)	0.95205 (7)	0.65234 (5)	0.01594 (13)	
H16A	0.4100	1.0040	0.6881	0.019*	
H16B	0.3434	0.9954	0.5925	0.019*	
C17	0.25587 (6)	0.94200 (7)	0.70697 (5)	0.01620 (13)	
H17A	0.2028	0.8949	0.6691	0.019*	
H17B	0.2700	0.8929	0.7645	0.019*	
C18	0.10673 (6)	1.04721 (7)	0.77555 (5)	0.01661 (13)	
H18A	0.1148	0.9920	0.8308	0.020*	
H18B	0.0580	1.0051	0.7307	0.020*	
C19	0.06022 (6)	1.17294 (8)	0.80442 (5)	0.01601 (13)	
H19A	0.0522	1.2281	0.7491	0.019*	
H19B	−0.0109	1.1594	0.8307	0.019*	
C20	0.23213 (6)	1.25503 (7)	0.83151 (5)	0.01593 (13)	
H20A	0.2805	1.2962	0.8772	0.019*	
H20B	0.2237	1.3121	0.7773	0.019*	
C21	0.28023 (6)	1.13121 (7)	0.80016 (5)	0.01553 (13)	
H21A	0.3497	1.1479	0.7716	0.019*	
H21B	0.2924	1.0762	0.8550	0.019*	
C22	0.08179 (6)	1.35398 (7)	0.90363 (5)	0.01556 (13)	
H22A	0.0054	1.3403	0.9144	0.019*	
H22B	0.0881	1.4162	0.8526	0.019*	
C23	0.13187 (6)	1.40919 (7)	0.99127 (5)	0.01650 (13)	
H23A	0.2097	1.4132	0.9847	0.020*	
H23B	0.1053	1.4960	1.0010	0.020*	
H1O1	0.1474 (12)	1.3609 (15)	1.1160 (11)	0.047 (4)*	

### Atomic displacement parameters (Å^2^)

**Table:**

	*U*^11^	*U*^22^	*U*^33^	*U*^12^	*U*^13^	*U*^23^
O1	0.0255 (3)	0.0212 (3)	0.0123 (2)	−0.0037 (2)	−0.0003 (2)	−0.00024 (19)
N1	0.0171 (3)	0.0124 (3)	0.0129 (2)	0.0007 (2)	−0.00349 (19)	−0.00178 (19)
N2	0.0140 (2)	0.0135 (3)	0.0123 (2)	−0.0022 (2)	−0.00079 (19)	−0.00190 (19)
N3	0.0138 (2)	0.0157 (3)	0.0119 (2)	−0.0006 (2)	−0.00102 (19)	−0.00231 (19)
C1	0.0180 (3)	0.0130 (3)	0.0113 (3)	0.0014 (2)	−0.0002 (2)	−0.0009 (2)
C2	0.0203 (3)	0.0175 (3)	0.0150 (3)	0.0033 (3)	−0.0019 (2)	−0.0009 (2)
C3	0.0276 (4)	0.0191 (3)	0.0159 (3)	0.0078 (3)	−0.0006 (3)	0.0010 (2)
C4	0.0352 (4)	0.0149 (3)	0.0196 (3)	0.0043 (3)	0.0037 (3)	0.0018 (3)
C5	0.0289 (4)	0.0155 (3)	0.0183 (3)	−0.0023 (3)	0.0036 (3)	−0.0002 (3)
C6	0.0199 (3)	0.0153 (3)	0.0131 (3)	−0.0010 (2)	0.0018 (2)	−0.0017 (2)
C7	0.0178 (3)	0.0205 (4)	0.0184 (3)	−0.0035 (3)	0.0021 (2)	−0.0039 (3)
C8	0.0157 (3)	0.0219 (4)	0.0184 (3)	0.0004 (3)	−0.0010 (2)	−0.0059 (3)
C9	0.0172 (3)	0.0165 (3)	0.0139 (3)	0.0036 (2)	−0.0019 (2)	−0.0036 (2)
C10	0.0251 (4)	0.0211 (4)	0.0160 (3)	0.0063 (3)	−0.0062 (3)	−0.0038 (3)
C11	0.0349 (4)	0.0213 (4)	0.0136 (3)	0.0087 (3)	−0.0013 (3)	−0.0003 (3)
C12	0.0306 (4)	0.0196 (4)	0.0171 (3)	0.0047 (3)	0.0058 (3)	0.0021 (3)
C13	0.0194 (3)	0.0177 (3)	0.0177 (3)	0.0023 (3)	0.0023 (2)	−0.0002 (2)
C14	0.0163 (3)	0.0136 (3)	0.0125 (3)	0.0029 (2)	−0.0007 (2)	−0.0020 (2)
C15	0.0172 (3)	0.0151 (3)	0.0172 (3)	0.0009 (2)	−0.0041 (2)	−0.0034 (2)
C16	0.0179 (3)	0.0134 (3)	0.0165 (3)	−0.0006 (2)	−0.0003 (2)	−0.0025 (2)
C17	0.0190 (3)	0.0126 (3)	0.0170 (3)	−0.0016 (2)	0.0007 (2)	−0.0017 (2)
C18	0.0168 (3)	0.0181 (3)	0.0150 (3)	−0.0054 (2)	0.0012 (2)	−0.0026 (2)
C19	0.0135 (3)	0.0210 (3)	0.0135 (3)	−0.0019 (2)	−0.0009 (2)	−0.0027 (2)
C20	0.0142 (3)	0.0159 (3)	0.0177 (3)	−0.0019 (2)	0.0001 (2)	−0.0039 (2)
C21	0.0144 (3)	0.0169 (3)	0.0152 (3)	−0.0003 (2)	−0.0026 (2)	−0.0037 (2)
C22	0.0169 (3)	0.0163 (3)	0.0135 (3)	0.0020 (2)	−0.0008 (2)	−0.0005 (2)
C23	0.0198 (3)	0.0161 (3)	0.0136 (3)	−0.0008 (2)	0.0009 (2)	−0.0015 (2)

### Geometric parameters (Å, °)

**Table:**

O1—C23	1.4221 (9)	C11—C12	1.3879 (13)
O1—H1O1	0.896 (16)	C11—H11A	0.9500
N1—C1	1.4284 (10)	C12—C13	1.3941 (11)
N1—C14	1.4327 (9)	C12—H12A	0.9500
N1—C15	1.4669 (9)	C13—C14	1.4002 (11)
N2—C21	1.4719 (9)	C13—H13A	0.9500
N2—C17	1.4750 (10)	C15—C16	1.5295 (10)
N2—C18	1.4766 (9)	C15—H15A	0.9900
N3—C20	1.4633 (9)	C15—H15B	0.9900
N3—C19	1.4641 (9)	C16—C17	1.5275 (11)
N3—C22	1.4644 (10)	C16—H16A	0.9900
C1—C2	1.3988 (10)	C16—H16B	0.9900
C1—C6	1.4109 (11)	C17—H17A	0.9900
C2—C3	1.3932 (11)	C17—H17B	0.9900
C2—H2A	0.9500	C18—C19	1.5160 (11)
C3—C4	1.3908 (13)	C18—H18A	0.9900
C3—H3A	0.9500	C18—H18B	0.9900
C4—C5	1.3900 (13)	C19—H19A	0.9900
C4—H4A	0.9500	C19—H19B	0.9900
C5—C6	1.4057 (11)	C20—C21	1.5168 (11)
C5—H5A	0.9500	C20—H20A	0.9900
C6—C7	1.4641 (11)	C20—H20B	0.9900
C7—C8	1.3446 (12)	C21—H21A	0.9900
C7—H7A	0.9500	C21—H21B	0.9900
C8—C9	1.4644 (12)	C22—C23	1.5163 (10)
C8—H8A	0.9500	C22—H22A	0.9900
C9—C10	1.4029 (11)	C22—H22B	0.9900
C9—C14	1.4072 (10)	C23—H23A	0.9900
C10—C11	1.3870 (13)	C23—H23B	0.9900
C10—H10A	0.9500		
			
C23—O1—H1O1	105.3 (10)	N1—C15—H15A	109.1
C1—N1—C14	114.65 (6)	C16—C15—H15A	109.1
C1—N1—C15	116.37 (6)	N1—C15—H15B	109.1
C14—N1—C15	116.79 (6)	C16—C15—H15B	109.1
C21—N2—C17	110.88 (6)	H15A—C15—H15B	107.8
C21—N2—C18	107.94 (6)	C17—C16—C15	112.28 (6)
C17—N2—C18	109.61 (6)	C17—C16—H16A	109.1
C20—N3—C19	107.69 (5)	C15—C16—H16A	109.1
C20—N3—C22	111.24 (6)	C17—C16—H16B	109.1
C19—N3—C22	110.21 (6)	C15—C16—H16B	109.1
C2—C1—C6	119.36 (7)	H16A—C16—H16B	107.9
C2—C1—N1	121.07 (7)	N2—C17—C16	113.42 (6)
C6—C1—N1	119.49 (6)	N2—C17—H17A	108.9
C3—C2—C1	121.06 (8)	C16—C17—H17A	108.9
C3—C2—H2A	119.5	N2—C17—H17B	108.9
C1—C2—H2A	119.5	C16—C17—H17B	108.9
C4—C3—C2	119.84 (8)	H17A—C17—H17B	107.7
C4—C3—H3A	120.1	N2—C18—C19	110.53 (6)
C2—C3—H3A	120.1	N2—C18—H18A	109.5
C5—C4—C3	119.69 (8)	C19—C18—H18A	109.5
C5—C4—H4A	120.2	N2—C18—H18B	109.5
C3—C4—H4A	120.2	C19—C18—H18B	109.5
C4—C5—C6	121.32 (8)	H18A—C18—H18B	108.1
C4—C5—H5A	119.3	N3—C19—C18	110.52 (6)
C6—C5—H5A	119.3	N3—C19—H19A	109.5
C5—C6—C1	118.73 (7)	C18—C19—H19A	109.5
C5—C6—C7	118.10 (7)	N3—C19—H19B	109.5
C1—C6—C7	123.14 (7)	C18—C19—H19B	109.5
C8—C7—C6	127.21 (7)	H19A—C19—H19B	108.1
C8—C7—H7A	116.4	N3—C20—C21	111.04 (6)
C6—C7—H7A	116.4	N3—C20—H20A	109.4
C7—C8—C9	125.70 (7)	C21—C20—H20A	109.4
C7—C8—H8A	117.1	N3—C20—H20B	109.4
C9—C8—H8A	117.1	C21—C20—H20B	109.4
C10—C9—C14	118.72 (7)	H20A—C20—H20B	108.0
C10—C9—C8	117.98 (7)	N2—C21—C20	111.61 (6)
C14—C9—C8	123.27 (7)	N2—C21—H21A	109.3
C11—C10—C9	121.56 (8)	C20—C21—H21A	109.3
C11—C10—H10A	119.2	N2—C21—H21B	109.3
C9—C10—H10A	119.2	C20—C21—H21B	109.3
C10—C11—C12	119.42 (7)	H21A—C21—H21B	108.0
C10—C11—H11A	120.3	N3—C22—C23	114.20 (6)
C12—C11—H11A	120.3	N3—C22—H22A	108.7
C11—C12—C13	120.12 (8)	C23—C22—H22A	108.7
C11—C12—H12A	119.9	N3—C22—H22B	108.7
C13—C12—H12A	119.9	C23—C22—H22B	108.7
C12—C13—C14	120.73 (8)	H22A—C22—H22B	107.6
C12—C13—H13A	119.6	O1—C23—C22	109.58 (6)
C14—C13—H13A	119.6	O1—C23—H23A	109.8
C13—C14—C9	119.38 (7)	C22—C23—H23A	109.8
C13—C14—N1	121.65 (7)	O1—C23—H23B	109.8
C9—C14—N1	118.91 (7)	C22—C23—H23B	109.8
N1—C15—C16	112.50 (6)	H23A—C23—H23B	108.2
			
C14—N1—C1—C2	116.85 (7)	C10—C9—C14—C13	−2.75 (11)
C15—N1—C1—C2	−24.50 (10)	C8—C9—C14—C13	175.33 (7)
C14—N1—C1—C6	−66.45 (9)	C10—C9—C14—N1	174.38 (7)
C15—N1—C1—C6	152.20 (7)	C8—C9—C14—N1	−7.54 (11)
C6—C1—C2—C3	−1.21 (11)	C1—N1—C14—C13	−111.94 (8)
N1—C1—C2—C3	175.49 (7)	C15—N1—C14—C13	29.25 (10)
C1—C2—C3—C4	0.74 (12)	C1—N1—C14—C9	71.01 (8)
C2—C3—C4—C5	0.03 (12)	C15—N1—C14—C9	−147.81 (7)
C3—C4—C5—C6	−0.32 (12)	C1—N1—C15—C16	−158.48 (6)
C4—C5—C6—C1	−0.14 (11)	C14—N1—C15—C16	61.01 (8)
C4—C5—C6—C7	−177.90 (7)	N1—C15—C16—C17	61.73 (8)
C2—C1—C6—C5	0.90 (10)	C21—N2—C17—C16	−67.12 (8)
N1—C1—C6—C5	−175.86 (7)	C18—N2—C17—C16	173.81 (6)
C2—C1—C6—C7	178.53 (7)	C15—C16—C17—N2	175.98 (6)
N1—C1—C6—C7	1.78 (10)	C21—N2—C18—C19	57.76 (8)
C5—C6—C7—C8	−150.54 (8)	C17—N2—C18—C19	178.62 (6)
C1—C6—C7—C8	31.80 (12)	C20—N3—C19—C18	60.33 (8)
C6—C7—C8—C9	3.66 (13)	C22—N3—C19—C18	−178.17 (6)
C7—C8—C9—C10	144.64 (8)	N2—C18—C19—N3	−61.60 (8)
C7—C8—C9—C14	−33.45 (12)	C19—N3—C20—C21	−58.73 (8)
C14—C9—C10—C11	2.62 (12)	C22—N3—C20—C21	−179.59 (6)
C8—C9—C10—C11	−175.56 (7)	C17—N2—C21—C20	−176.59 (6)
C9—C10—C11—C12	−0.88 (13)	C18—N2—C21—C20	−56.52 (8)
C10—C11—C12—C13	−0.73 (12)	N3—C20—C21—N2	58.74 (8)
C11—C12—C13—C14	0.55 (12)	C20—N3—C22—C23	−74.86 (8)
C12—C13—C14—C9	1.22 (11)	C19—N3—C22—C23	165.77 (6)
C12—C13—C14—N1	−175.83 (7)	N3—C22—C23—O1	−69.38 (8)

### Hydrogen-bond geometry (Å, °)

**Table:**

*D*—H···*A*	*D*—H	H···*A*	*D*···*A*	*D*—H···*A*
O1—H1O1···N2^i^	0.896 (16)	1.999 (16)	2.8822 (9)	168.3 (14)
C5—H5A···O1^ii^	0.95	2.41	3.3478 (11)	167.

Symmetry codes: (i) *x*, −*y*+5/2, *z*+1/2; (ii) *x*, −*y*+3/2, *z*−1/2.
